# Behavior‐dependent activity patterns of GABAergic long‐range projecting neurons in the rat hippocampus

**DOI:** 10.1002/hipo.22696

**Published:** 2017-02-02

**Authors:** Linda Katona, Ben Micklem, Zsolt Borhegyi, Daniel A. Swiejkowski, Ornella Valenti, Tim J. Viney, Dimitrios Kotzadimitriou, Thomas Klausberger, Peter Somogyi

**Affiliations:** ^1^ Department of PharmacologyUniversity of OxfordMansfield RoadOxfordOX1 3QTUK; ^2^ MRC Anatomical Neuropharmacology Unit, Department of PharmacologyUniversity of OxfordMansfield RoadOxfordOX1 3THUK; ^3^ MRC Brain Network Dynamics Unit, Department of PharmacologyUniversity of OxfordMansfield RoadOxfordOX1 3THUK; ^4^ Center for Brain Research, Medical University of ViennaViennaA‐1090Austria; ^5^ Department of BiochemistryEötvös Loránd UniversityBudapest1117Hungary; ^6^ Department of Neurophysiology and NeuropharmacologyCenter for Physiology and Pharmacology, Medical University of ViennaVienna1090Austria

**Keywords:** dendritic inhibition, theta oscillations, sharp wave‐ripples, somatostatin, CA1

## Abstract

Long‐range glutamatergic and GABAergic projections participate in temporal coordination of neuronal activity in distributed cortical areas. In the hippocampus, GABAergic neurons project to the medial septum and retrohippocampal areas. Many GABAergic projection cells express somatostatin (SOM+) and, together with locally terminating SOM+ bistratified and O‐LM cells, contribute to dendritic inhibition of pyramidal cells. We tested the hypothesis that diversity in SOM+ cells reflects temporal specialization during behavior using extracellular single cell recording and juxtacellular neurobiotin‐labeling in freely moving rats. We have demonstrated that rare GABAergic projection neurons discharge rhythmically and are remarkably diverse. During sharp wave‐ripples, most projection cells, including a novel SOM+ GABAergic back‐projecting cell, increased their activity similar to bistratified cells, but unlike O‐LM cells. During movement, most projection cells discharged along the descending slope of theta cycles, but some fired at the trough jointly with bistratified and O‐LM cells. The specialization of hippocampal SOM+ projection neurons complements the action of local interneurons in differentially phasing inputs from the CA3 area to CA1 pyramidal cell dendrites during sleep and wakefulness. Our observations suggest that GABAergic projection cells mediate the behavior‐ and network state‐dependent binding of neuronal assemblies amongst functionally‐related brain regions by transmitting local rhythmic entrainment of neurons in CA1 to neuronal populations in other areas. © 2016 The Authors Hippocampus Published by Wiley Periodicals, Inc.

## INTRODUCTION

Co‐ordinated activation of neuronal populations is required for the formation of representations in the cortex and for supporting behavior. Temporal neuronal coordination is reflected by rhythmic network activity recorded as local field potential oscillations. Oscillatory network states may arise through local interactions and/or through the interactions of different cortical areas mediated via long‐range glutamatergic and GABAergic projections (Amaral and Witter, 1989; Caputi et al., 2013). Abnormalities in the temporal co‐ordination of neuronal populations correlate with cognitive dysfunctions (Uhlhaas and Singer, 2006; Uhlhaas and Singer, 2012).

In the hippocampus, distinct rhythmic network states emerge in association with well‐defined behaviors (Vanderwolf, 1969; Buzsáki et al., 1983) segregating specific phases of mnemonic processing (Buzsáki, 1989; Hasselmo and McClelland, 1999). Theta frequency (5–12 Hz) oscillations regulate the plasticity of synaptic interactions during memory encoding and retrieval (Hyman et al., 2003; Hasselmo, 2005; Kwag and Paulsen, 2009; Douchamps et al., 2013). Moreover, synchronous neuronal population bursts during sharp wave associated ripple oscillations (SWRs) of 130–230 Hz may initiate plasticity in local and distant networks (Chrobak and Buzsáki, 1996; Sutherland and McNaughton, 2000; Girardeau et al., 2009; Maingret et al., 2016). Information is carried by the output of pyramidal cell assemblies entrained by rhythmic network states. Pyramidal cell activation is governed by glutamatergic inputs from cortical and subcortical areas (Amaral and Witter, 1989; Somogyi et al., 2014) and by neuromodulatory afferents originating in subcortical brain regions. Another major contributor to changes in pyramidal cell excitability is inhibition via subcellular‐domain specific GABAergic synaptic innervation. A diverse population of distinct GABAergic cell types (Somogyi, 2010) provide lamina specific inputs to different pyramidal cell compartments. For example, axo‐axonic cells target exclusively the axon initial segment, whereas basket cells innervate cell bodies and proximal dendrites.

The specialization of cortical GABAergic neurons is also reflected in their variable molecular composition and their differential origin and cell fate regulating programs (Fishell, 2007; Tricoire et al., 2011). One neuropeptide expressed by many GABAergic neurons is somatostatin (SOM)(Somogyi et al., 1984). The availability of SOM‐IRES‐Cre knock‐in mice (Taniguchi et al., 2011) made it possible to change the activity of the majority of SOM‐expressing neurons during behavioral tasks. It appears that a role of many SOM‐expressing interneurons is the regulation of dendritic excitability and signal integration via calcium dynamics (Gentet et al., 2012; Lovett‐Barron et al., 2012; Lovett‐Barron et al., 2014; Lovett‐Barron and Losonczy, 2014), including in a pilocarpine model of epilepsy (Peng et al., 2013). The inhibition of SOM‐expressing interneurons promotes burst firing (Gentet et al., 2012; Royer et al., 2012). Such experiments reveal some roles of SOM‐expressing neurons, but do not differentiate amongst functionally distinct SOM‐expressing neuronal types. In the rat hippocampal CA1 area, at least five distinct types of SOM‐expressing GABAergic neuron have been defined (Katona et al., 1999; Gulyás et al., 2003; Klausberger et al., 2004; Jinno et al., 2007; Melzer et al., 2012; Chittajallu et al., 2013). These innervate different proximo‐distal dendritic domains of pyramidal cells (Somogyi et al., 2014). There is limited information on the potential roles of identified types of SOM‐expressing neuron in controlling network activity. The activity of some SOM‐expressing interneuron types, e.g. bistratified and O‐LM cells, is cell type specific and network oscillatory state dependent (Varga et al., 2012, 2014; Katona et al., 2014). In addition to these exclusively locally terminating SOM‐expressing interneurons, a diverse population of long‐range SOM‐expressing GABAergic neurons send projections to the medial septum and/or retrohippocampal cortical areas (Gulyás et al., 2003; Jinno et al., 2007; Melzer et al., 2012). It has been suggested that such long‐range GABAergic afferents are well suited for the binding of different cortical areas into co‐operative networks (Buzsáki and Chrobak, 1995; Young and McNaughton, 2009).

We have tested if the activity of the long‐range projecting SOM‐expressing cells is similar to or different from the activity of exclusively local axon SOM‐expressing interneurons. We have analyzed in detail the contribution of rarely encountered GABAergic projection neurons to local network activity and predict how they participate in the functional binding of related brain areas.

## MATERIALS AND METHODS

### Experimental Subjects

All procedures involving experimental animals were performed in accordance with the Animals (Scientific Procedure) Act, 1986 (UK) and associated regulations at the University of Oxford and under the approval of the UK Home Office, of the Animal Care and Use Committees of the University of Oxford and of the Medical University in Vienna and of the Austrian Federal Ministry of Science and Research. Data reported are from 20 male Sprague‐Dawley rats (375–565 g) recorded between 8 a.m. and 8 p.m. Animals were housed in groups of two–four per cage in Oxford (*n* = 18 rats; 19–21C°; 55% humidity; reverse light/dark cycle, lights on from 8 pm–8 am) or Vienna (*n* = 2 rats, ZsB49 and O82; diurnal cycle, lights on from 6 am–6 pm). One to seven days before the experiments started, rats were housed in a cage on their own with *ad libitum* access to food pellets and water. During some recording sessions, the rats in Vienna received chocolate chip rewards.

### Surgical Procedures

Implantation of the head‐mounted recording setup, craniotomy and duratomy were performed mounted in a stereotaxic frame (Kopf Instruments) under isoflurane (IsoFlo, Abbott) anesthesia using analgesic (subcutaneous injection of 0.05 ml Rimadyl, Pfizer or Dipidolor, Janssen‐Cilag; 2 ml per 125 ml of drinking water provided for 48 h after surgery) and antibiotic (intraperitoneal injection of 0.1 ml 2.5% wt/vol Baytril, Bayer Vital) treatment, as reported in Katona et al (2014). Breathing was monitored visually by the experimenter and body temperature was maintained at 37°C with a heating pad connected to a homeothermic monitor (Harvard Apparatus).

After exposing and cleaning the skull, a cylindrical micro‐drive holder was attached above the left parietal cortex using dental acrylic (Refobacin R, Biomet). A main connector was placed above the frontal part of the skull, supported by five stainless steel screws attached to the bone. EEG and reference‐ground signals were connected to this head stage from one screw above the right prefrontal cortex (bregma −4 mm rostro‐caudal, bregma +2 mm medio‐lateral) and another above the cerebellum, respectively. All this was embedded in dental acrylic and was coated with blue light–polymerized cement (Tetric EvoFlow, Ivoclar Vivadent). Following at least three days of recovery, successive craniotomy and duratomy were completed above the right hemisphere under anesthesia. To prevent excessive tissue growth on subsequent days, 0.1 mg/ml of Mitomycin C was applied on the dura mater for 10 min between these two steps. Next, either a single wire electrode (50 μm tungsten, California Fine Wire; *n* = 14 rats) fixed on the skull or movable by a miniature drive (Haiss et al., 2010) or a microdrive movable tetrode (*n* = 6 rats) (Neuronelektrod Kft., Budapest, Hungary) was lowered into the hippocampus or cortex. While connected to the head stage, these electrodes and/or tetrodes were used to record local field potentials (LFPs). A layer of silicone (Kwik‐Sil, World Precision Instruments) was used to protect the cortical surface overnight and between recordings. During recordings, this was replaced by paraffin wax (Sigma‐Aldrich) for the ease of advancement of the glass electrode.

### In Vivo Extracellular Single Cell Recording and Juxtacellular Neurobiotin‐Labeling in Freely Moving Rats

Procedures were carried out as reported in Katona et al. (2014). Rats were anesthetized briefly by isoflurane and a miniature preamplifier (ELC mini‐preamplifier, NPI Electronic), two LED arrays and an accelerometer (Supertech Instruments; *n* = 14 rats) were connected to the head stage. A glass electrode (10–20 MΩ) filled with neurobiotin (Vector Laboratories Ltd, 1.5 or 3% wt/vol in 0.5 M NaCl) was advanced using a hydraulic (Narishige) or piezoelectric (Kleindiek Nanotechnik; (Lee et al., 2006)) microdrive to record single cell activity and LFPs in the hippocampus. Recordings commenced 1 h after recovery from anesthesia in either a darkened room (Oxford) or one with partially obscured windows (Vienna) using a recording arena (Oxford, *n* = 3 rats, 40 × 40 cm floor, 27 cm walls; or *n* = 15 rats, 50 × 50 cm floor, 27 cm walls; Vienna *n* = 2 rats, 40 × 60 cm floor, 30 cm walls) to which the rats were naive on the first day. Two video cameras captured the activity of the rats that were free to move around; one infra‐red sensitive for behavioral analysis and another one for position tracking using the head‐mounted LED arrays (Position Tracking System, v14.02.08, courtesy of K. Allen). Recordings were performed for 1–12 days (8 ± 3 d) after duratomy. After recording a neuron, the pipette was advanced towards the cell into a juxtacellular position and labeling using neurobiotin was attempted (Pinault, 1996). The stimulation was performed using ELC‐03M amplifier with active bridge compensation. When labeling was judged successful due to strong modulation, the pipette was retracted slowly and the neuron was left to recover from the entrainment. The rats were then briefly anesthetized in the stereotaxic frame using isoflurane while the recording equipment was removed. Following a post‐labeling period of up to 3 h the animals were deeply anesthetized using isoflurane followed by intraperitoneal injection of an overdose of Pentobarbital (JML Biopharm; 20% w/v solution; dose: 400 μl/100 g) and perfusion fixed (see below). If no stable neurons were found, sessions were finished by simply removing the recording devices and by covering the cortical surface with silicone between consecutive days.

### Recording Technical Specifications/Data Acquisition

Electrophysiological signals were amplified 1000× (BF‐48DGX and DPA‐2FS, NPI Electronic) and were digitized at 1 or 20 kHz (Power1401 A/D board, Cambridge Electronics Design). Measurements from the glass electrode were online band‐pass filtered according to three different frequency ranges (0.3 Hz–10 kHz, wide‐band; 0.3–500 Hz, LFP; 0.8–5 kHz, action potentials). Signals from the EEG and hippocampal/cortical electrodes or tetrodes were band pass filtered (0.3–300 Hz or 0.3 Hz–10 kHz). Elimination of 50 Hz noise without phase‐shift was provided by Hum Bugs (Quest Scientific Instruments). Accelerometer measurements were digitized at 1 or 20 kHz. Acquisition of all signals, except video tracking, went in parallel using Spike2 software (v7.06a, Cambridge Electronic Design, ced.co.uk).

### Behavioral State Detection and Electrophysiological Data Analyses

Following the criteria defined in Lapray et al. (2012), recording sessions have been segmented according to slow wave sleep, paradoxical sleep, quiet wakefulness and movement. In the recordings lacking accelerometer measurements LED array‐tracking data were imported into Spike2 for movement detection. We have defined sleep‐wake transitions as intervals at the end of slow wave sleep periods when the cortical LFP or EEG measured from a screw oscillates at low delta frequencies (<3 Hz) and is enriched in spindle oscillations (7–14 Hz) and, simultaneously, the hippocampal LFP oscillates at theta frequencies (5–12 Hz). Furthermore, we have detected theta oscillatory epochs (5–12 Hz) and SWRs (130–230 Hz) using Spike2 and MATLAB (Wavelet Toolbox, v7.9‐R2009b, MathWorks, uk.mathworks.com). Where possible, LFPs were analyzed from the implanted single wire electrodes and/or tetrodes. Otherwise, the detection was done on LFPs measured by the glass electrode in stratum oriens, where theta oscillations are in phase with those measured in the pyramidal cell layer and the power of ripple oscillations allowed identification of SWRs. Periods suggestive of the mechanical influence of the glass electrode on the firing of the recorded cell were excluded from further analyses.

For each cell, we have calculated average firing rates, point estimates (median ± interquartile range) for the inter‐spike interval distributions, autocorrelograms and the rate of occurrence of action potential bursts in the spike trains during different behavioral and oscillatory network states using MATLAB. From the last we have derived burst frequency and bursting probability indices using the formulas:

$$\frac{burstFrqMOV-\;burstFrqSWS}{burstFrqMOV+\;burstFrqSWS}$$

and

$$\frac{burstProbTHT-\;burstProbSWR}{burstProbTHT+\;burstProbSWR}$$

where burstFrqMOV and burstFrqSWS are the frequency of burst occurrence in Hz during movement and slow wave sleep, respectively; and burstProbTHT and burstProbSWR are the probability of bursting during theta oscillatory cycles and SWRs, respectively.

Furthermore, we quantified the depth of theta modulation of each cell using Rayleigh's method (Zar, 1999) and we computed the preferential mean theta phase of firing using normalized vector addition. During SWRs, we computed the median number of action potentials, we determined the average spiking probability histogram (5 ms bin width) during the ± 500 ms periods surrounding the peak of SWRs and created raster plots of the action potential firing aligned to the peak SWR power using colored lines delineating the beginnings and ends of aligned SWRs and surrounding SWR episodes. Sweeps were sorted according to when SWRs occurred, starting from the bottom with those during sleep and finishing with awake‐SWRs. Differences in SWR‐related firing rates versus firing rates outside SWR events were compared using two methods reported in Katona et al. (2014). In brief, the distribution and the mean of firing rates calculated for detected SWRs were compared with the distribution and the mean of 1000 surrogate firing rates representing the spiking of a given neuron outside detected SWRs. Two‐sample Kolmogorov‐Smirnov test with a probability of ≤0.05 indicated a significantly different firing rate distribution during detected SWRs from that calculated during outside SWR periods. The average of surrogate ‘SWRs’ and the 95% confidence intervals were plotted in solid black lines and in dashed black lines, respectively. Using these mean rates we have derived a SWR activation index using the formula:

$$\frac{rateSWRin-rateSWRout}{rateSWRin+rateSWRout}$$

### Anatomical Analyses and Single Cell Reconstruction

One to three hours after cell labeling, cardiac perfusion with saline was followed by ∼20 min fixation using 4% paraformaldehyde (wt/vol, Sigma‐Aldrich), 15% saturated picric acid (vol/vol, Sigma‐Aldrich), 0.05% glutaraldehyde (wt/vol, distilled grade, TAAB Laboratories Equipment Ltd) in 0.1 M phosphate buffer at pH ∼ 7.2. After brain removal, coronal sections were produced (70 μm nominal thickness, VT1000s vibratome, Leica Instruments) in the caudal to rostral direction. All procedures, including transmitted light, fluorescence and confocal microscopy were performed as reported in Katona et al. (2014). For immunohistochemistry indirect primary antibody detection method was used in combination with fluorochrome‐conjugated secondary antibodies. Immunoreactivity in the recorded cells was assessed visually and compared to neighboring cells not labeled by neurobiotin. A positive signal in the recorded cell was accepted if the subcellular location (e.g., plasma membrane), pattern, and strength of the signal were similar to that in non‐recorded cells. With the exception of calbindin, none of the molecules that were located in the recorded cells are known to be expressed by pyramidal neurons, which provided a within‐section negative control. Primary antibodies to the following molecules were used: CB (rabbit polyclonal, Swant #CB‐38, 1:5000, (Viney et al., 2013); mouse monoclonal, Swant #300, 1:400, (Tukker et al., 2013)); cannabinoid receptor type 1 (guinea pig and rabbit, gifted by Prof. M. Watanabe, Hokkaido University; both 1:1000, (Lasztoczi et al., 2011)); CR (goat polyclonal, Swant #CG1, 1:1000, (Unal et al., 2015)); GABA‐A receptor subunit alpha‐3 (guinea pig polyclonal, 1:4000, (Fritschy and Mohler, 1995)); M2R (rat, Millipore # MAB367, 1:250, (Ferraguti et al., 2005)); mGluR1a (guinea pig and goat, gifted by Prof. M. Watanabe, FRONTIER INSTITUTE; 1:500 and 1:750 or 1000, respectively; (Katona et al., 2014)); NOS (mouse polyclonal, Sigma‐Aldrich #N2280, 1:1000, (Lapray et al., 2012); rabbit polyclonal, Millipore #AB5380, 1:1000, (Viney et al., 2013)); neuropeptide Y (rabbit polyclonal, ImmunoStar #22940, 1:700 or 5000, (Katona et al., 2014); sheep polyclonal, Millipore #AB1583, 1:500, (Viney et al., 2013)); PV (guinea pig polyclonal, Synaptic Systems #195 004; mouse monoclonal, Swant #235; both 1:5000, (Katona et al., 2014)); reelin (mouse monoclonal, Millipore #MAB5364, 1:1000, (Unal et al., 2015)); SOM (mouse monoclonal, GeneTex #GTX71935, 1:200, 500 or 600, (Katona et al., 2014)); vasoactive intestinal polypeptide (mouse monoclonal, gifted by Dr. G. Ohning, UCLA, 1:50000, (Lapray et al., 2012)). Secondary antibodies were raised in donkey against immunoglobulin G of the species of origin of the primary antibodies and conjugated to Alexa 405 (1:100 or 250); DyLight405 (blue, 1:100, 200, 250 or 400); Alexa 488 (1:1000); cyanine 3 (Cy3, 1:400); DyLight594 (red, 1:500); cyanine 5 (Cy5, 1:250) and DyLight649 (infra‐red, 1:250 or 400). See dilutions used in brackets. The donkey‐anti‐rabbit‐Alexa405 and donkey‐anti‐mouse‐Alexa488 were purchased from Invitrogen. Secondary antibodies conjugated to DyLight405, Cy3, DyLight594, Cy5 and DyLight649 fluorophores were purchased from Stratech.

Three labeled projection cells were selected for reconstruction. Two neurons (D43j and ZsB49b) were digitally reconstructed in 3D from resin‐embedded, osmium‐treated serial coronal cut sections reacted for HRP using Neurolucida (MBF Bioscience), a Nikon Eclipse 80i transmitted light microscope and Lucivid microdisplay (MBF Bioscience) in continuous mode using a VC Plan Apo 100x/1.4 oil immersion objective. One neuron (D29o) was manually traced using a microscope‐attached drawing tube using a NPL Fluotar 40x/0.7 and a Pl Apo 63x/1.4 oil immersion objective.

## RESULTS

**Figure 1:**
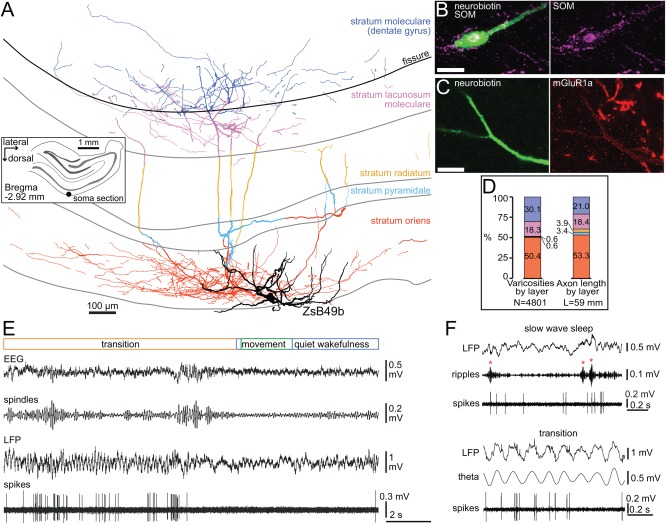
Behavior related activity of a novel back‐projecting non‐pyramidal cell (ZsB49b) in the hippocampal CA1 area. (A) Reconstruction of the labeled neuron. Note the target layer selectivity of the axon (color‐coded by layers) innervating strongly strata oriens and lacunosum moleculare, crossing the hippocampal fissure and forming a wide axonal plexus in the outer third part of dentate molecular layer. The soma and horizontally oriented dendrites (black) were located in stratum oriens. *Inset*, location of the cell body (black dot) in stratum oriens in a coronal section, left hemisphere. Shaded gray areas delineate the pyramidal and granule cell layers. (B) The neurobiotin‐labeled cell body and a proximal dendrite (green) were immunopositive for somatostatin (confocal microscopic image, maximum intensity projection, z stack, height 14 μm). Scale bar, 10 μm. (C) The dendritic membrane of the labeled neuron was enriched in the metabotropic glutamate receptor type 1 alpha (single plane image). Scale bar, 10 μm. (D) Proportions of axon varicosities and axon length quantified by target layers, showing preference for stratum oriens and a significant back‐projection to the molecular layer (color coding as in A). (E) Recording trace of the activity pattern of this neuron; when the rat roused the cell became inactive for a few seconds. (F) *Top*, Recording trace of the activity of the neuron during slow wave sleep enriched in hippocampal SWRs (red asterisks). *Bottom*, a transition period towards arousal with prominent hippocampal theta oscillations. Spindles, filtered 7–14 Hz; ripples, filtered 130–230 Hz; theta, filtered 5–12 Hz.

**Figure 2:**
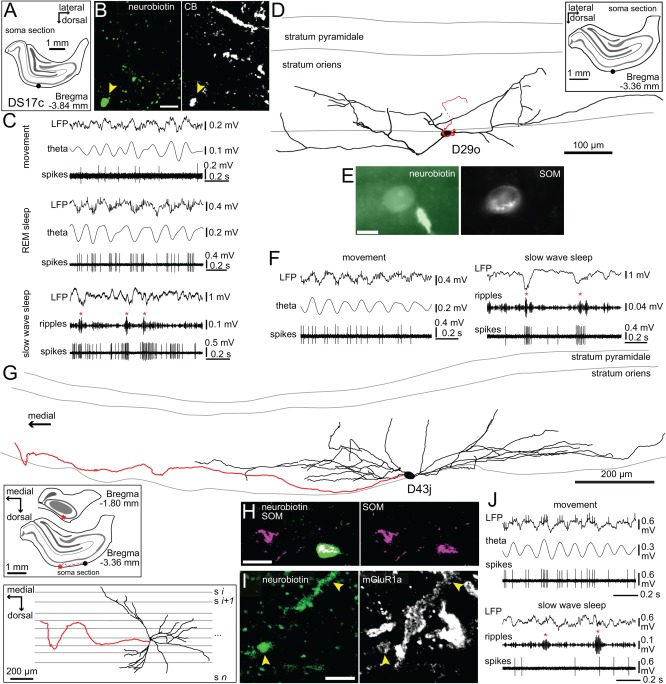
Behavior related activity of horizontal SOM‐expressing non‐pyramidal cells (DS17c, D29o and D43j) in the hippocampal CA1 area. (A) The location of the cell body of DS17c (black dot) was extrapolated from the convergence of dendrites in a coronal section of the right hemisphere. Shaded gray areas delineate the pyramidal and granule cell layers also in subsequent insets. (B) The neurobiotin filled dendrite (arrowhead) was immunopositive for calbindin (confocal microscopic single plane image). Scale bar, 5 μm. (C) Recording traces of the activity of DS17c during movement and REM sleep with prominent theta oscillations and during slow wave sleep with hippocampal SWRs (red asterisks). (D) Reconstruction of the neuron D29o with soma and the dendritic tree (four 70‐μm‐thick sections) in stratum oriens and the axon originating from a proximal dendrite and projecting caudally towards the subiculum; it could be followed through ten sections from the soma spanning ∼0.7 mm where the labeling faded. *Inset*, location of the cell body (black dot) in stratum oriens of the right hemisphere. (E) The neurobiotin‐filled cell body (green) was immunopositive for somatostatin (single plane epifluorescence image). Scale bar, 10 μm. (F) Recording traces of the activity of D29o during movement with prominent theta oscillations and during slow wave sleep enriched in hippocampal SWRs. (G) Reconstruction of the cell body, extensive dendritic tree (black, ten 70‐μm‐thick sections) and the initial portion of the main axon (red, four 70‐μm‐thick sections) of cell D43j. The projection axon travelled in rostro‐medial direction and was last observed in the anterior tip of the hippocampus (see top inset) before the signal faded. *Inset top*, location of the soma (black dot) in stratum oriens and of the projection route (red asterisk) of the axon, right hemisphere. *Inset bottom*, reconstruction viewed from top of the brain. (H) The neurobiotin‐labeled soma (green) was immunopositive for somatostatin (confocal maximum intensity projection, z stack, height 4.2 μm). Scale bar, 20 μm. (I) Weak immunoreactivity for metabotropic glutamate receptor type 1 alpha was localized in the labeled dendrites located amongst strongly immunopositive neurons (white, confocal maximum intensity projection, z stack, height 3.6 μm). Scale bar, 5 μm. (J) Recording traces of the activity of D43j during movement with prominent theta oscillations and during slow wave sleep with hippocampal SWRs. Theta, filtered 5–12 Hz; ripples, filtered, 130–230 Hz.

We have determined the activity of hippocampal neurons using a previously established combination of techniques in freely moving rats (Lapray et al., 2012; Katona et al., 2014). In brief, we first recorded extracellularly the action potential discharge of individual neurons using a glass pipette during both wakefulness, which included periods of movement, and different stages of sleep. Subsequently we moved the pipette into juxtacellular position and attempted to label the recorded cell with neurobiotin. When successful, the labeling allowed the post‐hoc identification of the cell type using immunohistochemistry and neuronal reconstruction. Here, we report the behavior dependent temporal specialization of identified long‐range projecting GABAergic neurons (*n* = 7) in stratum oriens of the hippocampal CA1 area (Figs. 1A and 2A,D,G), together with a set of unlabeled putative GABAergic cells (*n* = 25) recorded in the same hippocampal area. Unlabeled neurons were included into the dataset only if the amplitude of their action potentials was above the threshold for automatic spike detection and if the recorded periods of cell activity included different behavioral states of at least 2 s duration. The group of unlabeled interneurons recorded in stratum oriens may include O‐LM cells (McBain et al., 1994; Maccaferri et al., 2000; Varga et al., 2012; Katona et al., 2014), horizontal bistratified, basket and axo‐axonic cells (McBain et al., 1994; Sik et al., 1995; Maccaferri et al., 2000; Losonczy et al., 2002; Ganter et al., 2004; Varga et al., 2014) and various long‐range projecting GABAergic cell types (Sik et al., 1995, 1994; Gulyás et al., 2003; Jinno et al., 2007). Trilaminar cells, a distinct type of projection neuron (Sik et al., 1995; Ferraguti et al., 2005), are not present in this sample, as their prolonged high frequency (>200 Hz) bursts of action potentials is a unique feature of their firing pattern. We then compared the temporal patterns of firing of hippocampal SOM+ projection cells with that of SOM+ local targeting bistratified and O‐LM cells (Katona et al., 2014). Together with dendrite‐targeting O‐LM and bistratified cells (Varga et al., 2012, 2014; Katona et al., 2014; Lovett‐Barron and Losonczy, 2014), projection cells may regulate signal integration in local CA1 pyramidal cells (Pouille and Scanziani, 2004; Lovett‐Barron et al., 2014) via influencing dendritic calcium dynamics (Spruston et al., 1995).

### Long‐Range Projecting GABAergic Non‐Pyramidal Neurons Identified in the CA1 Area of Freely Moving Rats

Six out of seven recorded hippocampal cells (DS17c, D29o, D43j, DK02Ag, O82c, and TV09u) showed dendritic and axonal arborization and molecular expression profiles reported previously for GABAergic long‐range projecting cells in the CA1 area (Jinno et al., 2007); one neuron (ZsB49b) had novel features (Fig. 1A–D).

Somata (*n* = 2/6) and horizontal dendrites (*n* = 6/6) were found in stratum oriens and at least one thick projection axon in each brain (*n* = 6/6) identified the neurons as projection cells (Fig. 2A,B,D,E,G–I). The local axon collaterals of the majority of projection cells form synapses with basal and stratum radiatum oblique dendrites of pyramidal cells, and to lesser extent GABAergic interneurons (Jinno et al., 2007; Takács et al., 2008). The labeling quality varied from full labeled (ZsB49b; Fig. 1A) to only the axon labeled; we have reconstructed the complete dendritic tree and the initial part of the axon of two cells (D29o, D43j; Fig. 2D,G). Where possible, the projection axons were followed through several coronal sections in either the caudal (*n* = 3/6, D29o, DK02Ag, O82c) or rostral direction (*n* = 2/6, D43j, TV09u) until the 3,3′‐diaminobenzidine (DAB) signal has faded. Due to suboptimal labeling, no termination target areas could be identified, but based on previous reports and the caudo‐medial direction of travel of some visualized axons (*n* = 3/6), subiculum was one likely target area (Jinno et al., 2007). Most projection axons (*n* = 5/6) travelled within stratum oriens or at the border with alveus and the cortical white matter. The axon that was followed for the longest (D43j; Fig. 2G) travelled for a distance of ∼1.5 mm from the soma (21 sections of ∼70 μm thickness) in the rostro‐medial direction and was last observed in the anterior tip of the dorsal hippocampus before the DAB‐signal has faded, likely heading towards the medial septum. The axon of another cell (DK02Ag) branched in stratum oriens into a radial collateral which travelled to the strata radiatum/lacunosum moleculare border where it turned parallel with the border and continued in the caudal direction for ∼840 μm (twelve ∼70 μm thick sections) after which the signal could no longer be detected. Such a projection route has been observed for projection cells in the CA1 targeting the subiculum (Jinno et al., 2007). Some neurons were tested for the expression of known GABAergic projection cell molecular markers. Two out of five cells were immunopositive for the neuropeptide somatostatin (SOM; Fig. 2E,H), in the other three cases we were only able to test dendrites which proved immunonegative. Some long‐range projecting SOM‐expressing GABAergic neurons in the mouse hippocampus have wide‐spread axonal distribution innervating the medial septum and are derived from early‐born GABAergic ‘operational hub neurons’ (Picardo et al., 2011; Villette et al., 2016). The dendritic membrane of one out of three tested cells was enriched in metabotropic glutamate receptor type 1 alpha (mGluR1a; Fig. 2I). The dendrites of one cell were immunopositive for the calcium binding protein calbindin (CB; Fig. 2B); two other tested cells were immunonegative. One projection cell (D43j) co‐expressing SOM and mGluR1a was also immunopositive for the secreted extracellular matrix protein reelin. Two tested cells were found immunonegative for muscarinic acetylcholine receptor M_2_ (M2R) and the calcium binding protein parvalbumin (PV), one of which was also immunonegative for both nitric oxide synthase (NOS) and the calcium binding protein calretinin (CR).

The seventh recorded neuron was visualized and reconstructed completely (ZsB49b; Fig. 1A). This cell had similar cell body location and horizontally oriented dendrites at the border between stratum oriens and alveus like the other projection cells but its axonal arborization pattern was novel. The lack of a single thick projection axon and the extensive local axon collateral system in stratum lacunosum moleculare were suggestive of an O‐LM cell. However, in contrast to O‐LM cells, the axon of this neuron had a very extensive arborization in stratum oriens and crossed the hippocampal fissure forming a dense axonal plexus also in the molecular layer of the dentate gyrus (DG). We found strata oriens, lacunosum moleculare and moleculare of DG to be most heavily innervated by the neuron when quantified as total axonal length and total number of varicosities per layer (Fig. 1D), in contrast to strata radiatum and pyramidale, which had very few boutons. Such target layer selectivity and the existence of a back‐projection from the CA1 area to the DG of a single cell have not been reported before, to our knowledge. In order to identify a likely subpopulation of such neurons within the hippocampus, we attempted to determine a combination of known GABAergic cell molecular markers that may be uniquely expressed in this novel cell type. After an extensive series of immunohistochemical tests, we have found that the cell body and the dendrites of the neuron were immunopositive for two markers SOM and mGluR1a (Fig. 1B,C). The neuron was immunonegative for PV, CB, CR, NOS, M2R, GABA‐A receptor subunit alpha‐3, neuropeptide Y, vasoactive intestinal polypeptide (VIP), and cannabinoid receptor type 1, the last three tested on segments of the axon. The results of the tests failed to identify a subpopulation of neurons similar to ZsB49b because the two positive markers, SOM and mGluR1a, are also commonly expressed by long‐range projecting GABAergic cells and local axon O‐LM cells, but the lack of a long‐range projection axon and the dentate‐innervating axonal collaterals clearly differentiate it from the former and latter cells, respectively.

### GABAergic Projection Cell Activity Patterns in the Hippocampal CA1 Area of Freely Moving Rats

**Figure 3:**
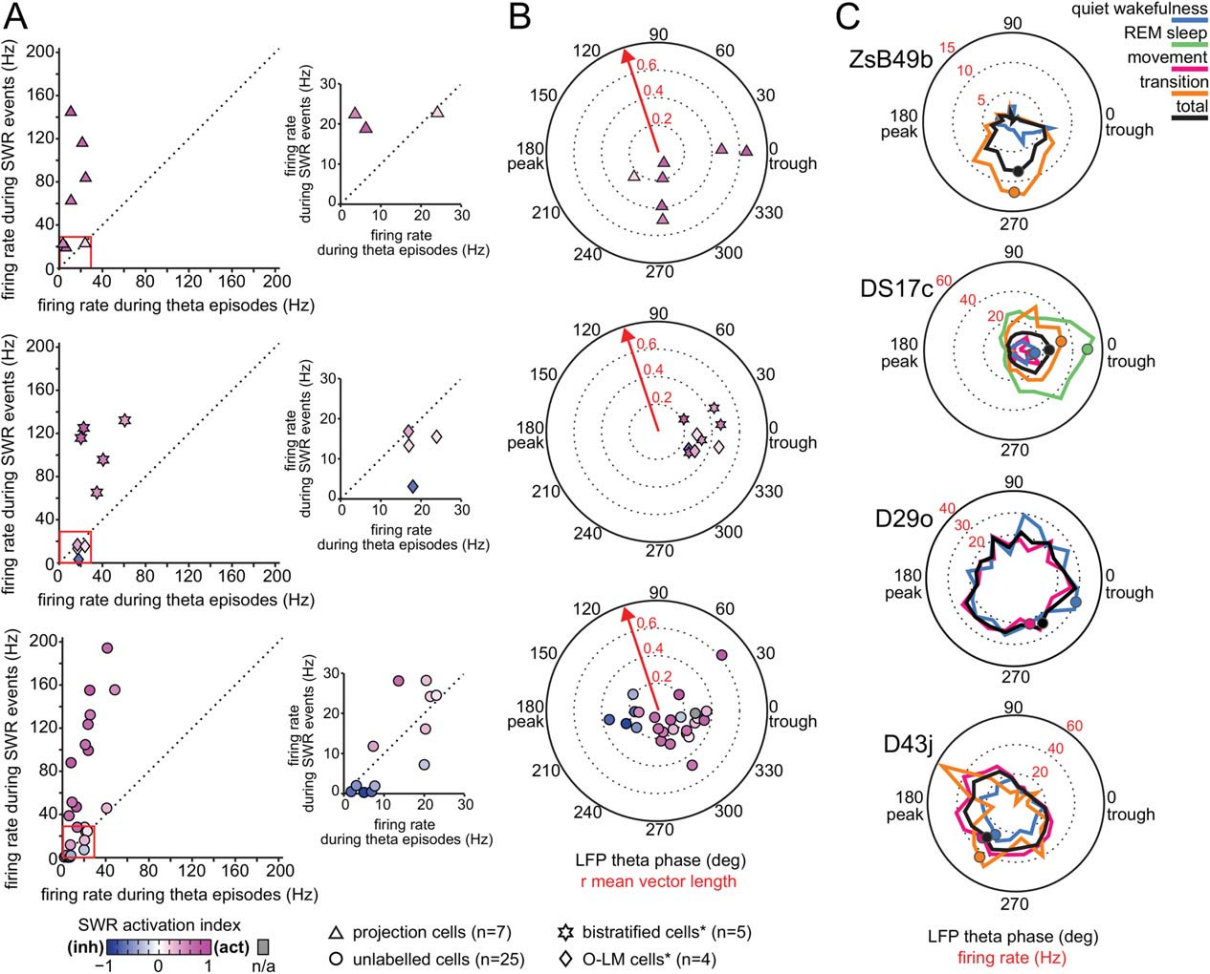
Comparison of oscillatory network state dependent firing rate and spike timing of hippocampal non‐pyramidal cells. (A) *Left*, comparison of the firing rate of individual neurons recorded in stratum oriens and at the border between strata oriens/pyramidale during theta episodes and SWR events (different symbols used for neuron categories). *Right*, enlarged firing rate range of 0–30 Hz (red box) for clarity. Note that the majority of cells, including projection cells and bistratified cells, but not O‐LM cells, fired at higher rates during SWR events than during theta episodes. Color coding of symbols represents the SWR‐related activity of each neuron (see methods). Note high SWR activation indices for the majority of neurons (shades of magenta) demonstrating strong increases in firing rates occurring during SWRs as compared to periods outside SWRs. *, data taken from Katona et al. 2014 for comparison. (B) Preferred mean firing phases of individual neurons during theta oscillations and the strength of their spike coupling to theta cycles (dotted circles along red arrow). *Top*, note that five out of seven labeled projection cells increased their firing rate during the descending slope of theta oscillatory cycles (around 270 deg); two neurons were strongly coupled to the trough of theta cycles, similar to bistratified and O‐LM cells. As a group, most recorded neurons increased their firing rates at and around the trough of theta cycles. Same symbols and color coding used as in A. *, data taken from Katona et al. 2014 for comparison. (C) Distribution of firing phases (color‐coded by behavioral states) and associated firing rates (red numbers) of the labeled GABAergic projection neurons during theta frequency oscillations. Mean preferential theta phases (colored dots) plotted only for theta periods when the cells were significantly coupled to theta oscillatory cycles. Note strong tuning of three of the neurons and the broad distribution of firing phases and associated firing rates for cell D29o.

**Figure 4:**
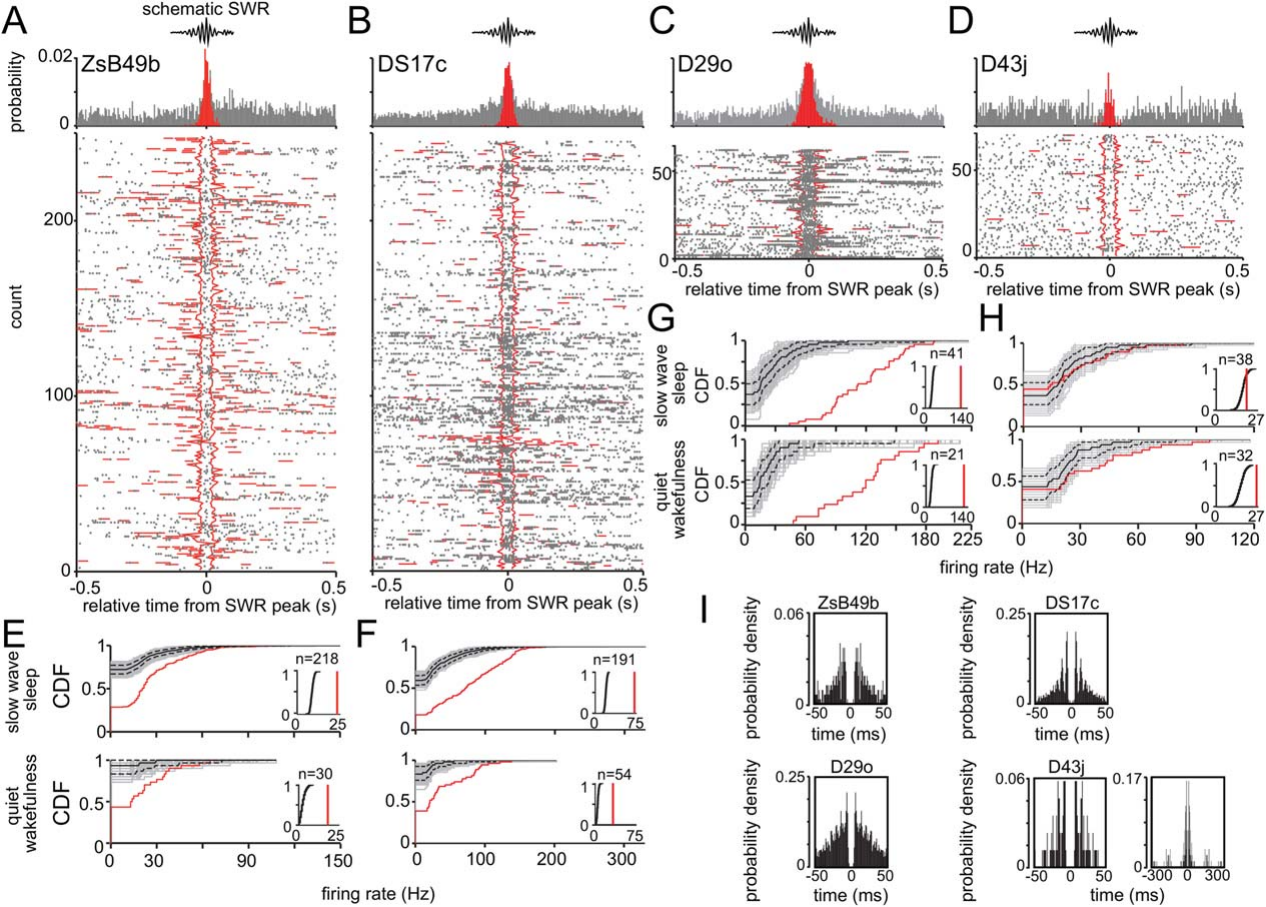
Activity of labeled GABAergic projection cells during SWRs. (A–D) Average firing probability densities and raster plots of the labeled neurons during SWRs (red bars in histogram) compared to the ± 0.5 s surrounding the peak of SWRs (gray bars in histogram). Most neurons fired with higher probability during SWRs compared to the ± 0.5 s surrounding the peak of SWR events. Raster plots were aligned to the peak SWR‐power. Red lines delineate the beginnings and ends or the duration of ripple events outside the reference SWRs. (E–H) Distribution of SWR‐related firing rates displayed as cumulative distribution functions (CDFs) during sleep and wakefulness. Surrogate sets of 1000 firing rate‐distributions are shown (gray; median, solid black; 95% confidence intervals, dashed lines). *Insets*, comparison of mean SWR‐related firing rates (red lines) with the distribution of mean SWR‐related rates (black lines) from the surrogate sets. (E–G) The measured distribution of firing rates (red) was significantly right‐shifted from the median (black) of the surrogate set demonstrating strong increases in activity during SWR events over the full range of firing rates (cells ZsB49b, DS17c and D29o; *P* < 0.05; two‐sample KS test, for both states) or when comparing mean SWR‐related rates (*P* < 0.05, relative to the surrogate CDF, for both states). (H) During sleep, the measured rates of D43j (red) were similar to those expected from the surrogate distributions (*P* = 0.4; two‐sample KS test). During wakefulness, the measured distribution of firing rates (red) was significantly right‐shifted (*P* < 0.05; two‐sample KS test) from the median of the surrogate set (black). *Insets*, the mean SWR‐related firing rate (red line) was higher than expected from the distribution of surrogate mean SWR‐related rates (black line) during both, sleep and wakefulness (*P* < 0.05, relative to the surrogate CDF). (I) Spikes triggered autocorrelograms during SWR events. Note short interval peaks demonstrating ripple‐rhythmic spiking of some neurons. Time axes were limited to 50 ms and 300 ms, bin width was set to 1 and 3 ms, respectively.

**Figure 5:**
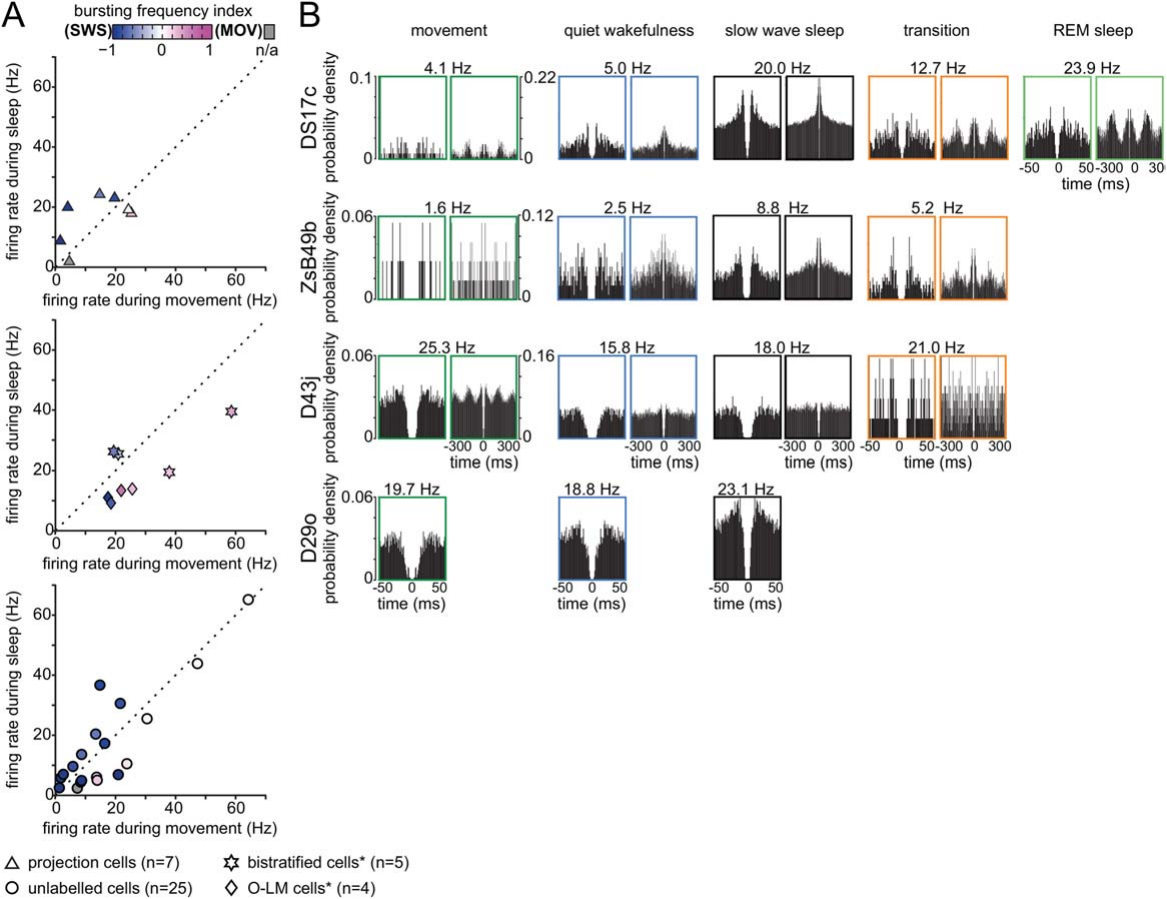
Behavioral state dependent action potential firing rate and pattern of *in vivo* recorded non‐pyramidal cells in stratum oriens. (A) Changes in firing rate of individual neurons recorded in stratum oriens and at the border between strata oriens/pyramidale during behavior (different symbols show neuron categorization). Note the wide range in the behavior‐related firing rates of neurons. *Top*, four out of seven labeled projection cells were more active during sleep than during movement. Color coding represents changes in bursting frequency during sleep and movement (see methods). *, data taken from Katona et al. 2014 for comparison. (B) Behavioral state specific action potential autocorrelograms of the labeled GABAergic projection neurons show great variation; mean firing rates are shown on top. Time axes were limited to 50 ms (left) and 300 ms (right), bin width was set to 1 and 3 ms, respectively.

Co‐ordinated activation of large groups of neuron in the hippocampus is reflected in the local field potential by rhythmic neuronal network events associated with well‐defined behaviors. Rhythmic activity at theta frequencies (5–12 Hz) is prominent during movement, quiet wakefulness and rapid eye movement (REM) sleep, whereas high frequency (130–230 Hz) short duration SWRs occur frequently during slow wave sleep, immobility and consummatory behavior. We have recorded and analyzed the action potential discharge of individual GABAergic neurons during these two oscillatory network events and corresponding behavioral states (Figs. 1E,F, 2C,F,J and 3–5).

### Firing Patterns of GABAergic Projection Neurons during Rhythmic Network States

During theta oscillations, the seven labeled projection cells fired with mean rates ranging between 3.6 Hz and 24.5 Hz (Fig. 3A and Table 1). Similar high variability was found in the mean firing rates of the unlabeled neurons (*n* = 25) with a range between 1.8 Hz and 48.4 Hz (Table 1), which was mirrored by variability in the inter spike interval (ISI) distributions (Table 2). As a group, most recorded neurons (*n* = 24/32) fired with lower mean rates during theta epochs than during SWRs, similar to bistratified cells but unlike O‐LM cells (Fig. 3A and (Katona et al., 2014)). This included six out of seven labeled projection cells. Seven cells had higher mean firing rates during theta epochs than during SWRs, two of which were similar to O‐LM cells (Fig. 3A and (Katona et al., 2014)). Differences in firing rates were paralleled by differences in the inter spike interval distributions, with median ISI lengths being, on average, six times shorter (8.8 ± 3.6 ms vs 49.3 ± 34.6 ms) during SWRs than during theta epochs (Table 2).

**Table 1:** Firing Rates and Patterns of GABAergic Neurons in Stratum Oriens

	Mean firing rate (Hz)									
Cell	Movement	Slow wave sleep	Quiet wakefulness	Sleep‐wake transition	Theta episodes	SWR events	Mean theta phase of firing	Mean theta vector length of firing	SWR activation index^a^	Median No spikes per SWR
D29o^b^	19.7	23.1	18.8	n/a	21.4	116.2	304.6	0.09	0.71	7
D43j^c^	25.3	18.0	15.8	21.0	24.1	23.0	226.8	0.24	0.16	1
DK02Ag	14.7	24.3	11.3	n/a	10.8	144.8	0.6	0.65	0.76	7
DS17c*^d^	4.1	20.0	5.0	12.7	11.0	62.7	2.7	0.47	0.61	2
O82c	24.3	19.2	16.2	n/a	24.5	83.7	282.8	0.19	0.63	8
TV09u	4.6	1.9	4.5	n/a	6.3	19.1	275.5	0.39	0.72	1
ZsB49b^e^	1.6	8.8	2.5	5.2	3.6	22.7	275.1	0.49	0.53	1
Mean firing rate during rapid eye movement sleep: *, 23.9 Hz; n/a, not applicable.										
LK06o	47.3	43.9	41.7	n/a	41.5	194.2	278.3	0.22	0.66	12
LK10x	23.8	10.5	17.3	n/a	21.5	24.2	349.8	0.30	0.21	1
LK13f*	16.4	17.3	17.1	n/a	20.5	28.2	342.6	0.29	0.24	1
LK14e	26.9	n/a	20.2	n/a	26.6	n/a	356.4	0.28	n/a	n/a
LK14k	1.3	2.5	2.1	2.2	1.8	0.5	182.2	0.16	−0.69	0
LK14l**	1.8	5.8	2.8	3.4	5.0	0.3	202.6	0.24	−0.89	0
LK14p***	2.6	7.0	4.1	3.0	3.1	2.0	219.7	0.19	−0.52	0
LK14r	7.3	2.4	6.5	8.3	6.9	0.5	190.5	0.35	−0.73	0
LK14s	13.7	6.0	9.6	n/a	12.8	47.0	40.7	0.62	0.79	2
LK14t	8.4	4.4	5.0	n/a	7.7	1.9	144.9	0.21	−0.40	0
LK18d	8.7	4.9	7.7	4.5	7.3	11.8	322.8	0.27	0.38	0
LK18h	n/a	10.3	19.7	n/a	20.0	7.2	345.2	0.18	−0.26	0
LK18x	n/a	19.2	22.7	n/a	23.0	24.5	321.0	0.30	0.10	1
LK20g	22.9	n/a	16.2	n/a	23.9	99.4	348.9	0.35	0.74	5
LK20l	14.8	36.7	17.0	n/a	25.6	132.3	286.7	0.16	0.59	9
LK22b	21.6	30.6	22.2	n/a	21.2	104.7	274.9	0.13	0.63	5
LK25b	47.0	n/a	25.4	n/a	40.9	45.9	359.6	0.36	0.29	2
LK25h	8.7	13.6	7.8	n/a	7.9	88.0	36.1	0.20	0.79	6
LK26b	13.9	5.0	7.1	n/a	13.6	28.1	326.5	0.26	0.64	1
LK27a	13.4	20.4	6.2	n/a	8.9	51.4	303.1	0.47	0.61	3
D25i	64.2	65.2	42.5	n/a	48.4	155.6	184.2	0.13	0.48	9
D38j	25.9	n/a	25.8	n/a	23.7	123.5	252.8	0.05	0.68	7
DS20k	30.5	25.5	24.0	n/a	25.2	155.1	326.6	0.12	0.76	8
DS20p	5.8	9.6	7.1	7.2	6.0	38.8	292.1	0.26	0.63	2
DS20z	20.9	6.9	18.0	18.2	20.3	16.1	311.6	0.18	0.29	0
Mean firing rate during rapid eye movement sleep *, 24.9 Hz; **, 10.7 Hz; ***, 3.1 Hz; n/a, not applicable.										

a see Methods.

Molecular expression profiles (+, immunopositive; ‐, immunonegative).

b SOM+/mGluR7a‐.

c SOM+/mGluR1a+/reelin+/CB‐/M2R‐/PV‐/NOS‐/CR‐.

d CB+/mGluR1a‐/M2R‐/PV‐.

e SOM+/mGluR1a+/CB‐/M2R‐/PV‐/NOS‐/CR‐/NPY‐/VIP‐/GABAAa3‐/CB1R‐.

**Table 2:** Point Estimates of ISI Distributions of GABAergic Neurons in Stratum Oriens

	Median ± interquartile range of ISI (ms)					
Cell	Movement	Slow wave sleep	Quiet wakefulness	Sleep‐wake transition	Theta episodes	SWR events
D29o	34.2 ± 38.6	20.4 ± 31.1	31.0 ± 43.5	n/a ± n/a	33.1 ± 33.4	6.6 ± 2.6
D43j	25.5 ± 34.1	38.0 ± 49.0	42.7 ± 54.3	29.8 ± 62.8	26.6 ± 36.8	12.0 ± 8.8
DK02Ag	29.1 ± 110.7	10.6 ± 23.6	16.7 ± 62.2	n/a ± n/a	108.5 ± 126.9	6.3 ± 1.9
DS17c*	155.3 ± 222.8	13.4 ± 35.3	59.8 ± 185.7	26.0 ± 100.6	30.9 ± 113.7	6.6 ± 2.2
O82c	19.3 ± 33.0	15.4 ± 39.7	27.8 ± 56.8	n/a ± n/a	20.4 ± 32.5	6.6 ± 3.8
TV09u	101.8 ± 129.5	270.3 ± 355.4	111.3 ± 149.3	n/a ± n/a	100.1 ± 104.4	16.3 ± 6.8
ZsB49b	83.2 ± 150.1	36.7 ± 96.3	45.4 ± 200.3	75.4 ± 152.7	88.5 ± 167.7	12.9 ± 9.9
Median ± interquartile range of ISI during rapid eye movement sleep: *, 20.4 ± 37.9 ms; n/a, not applicable.						
LK06o	13.5 ± 15.3	10.2 ± 13.4	11.8 ± 17.7	n/a ± n/a	14.5 ± 18.1	4.4 ± 2.6
LK10x	31.9 ± 29.9	56.8 ± 75.1	39.3 ± 43.9	n/a ± n/a	34.7 ± 34.0	6.8 ± 5.2
LK13f*	42.3 ± 49.3	36.6 ± 65.2	38.0 ± 57.0	n/a ± n/a	35.4 ± 37.7	7.9 ± 4.9
LK14e	25.9 ± 25.7	n/a ± n/a	31.5 ± 38.8	n/a ± n/a	25.9 ± 26.2	n/a ± n/a
LK14k	189.8 ± 460.3	109.3 ± 454.0	193.4 ± 438.5	25.6 ± 162.7	107.3 ± 381.1	11.3 ± 13.3
LK14l**	169.4 ± 370.9	60.4 ± 147.1	166.9 ± 321.5	112.9 ± 191.4	73.2 ± 127.2	n/a ± n/a
LK14p***	105.4 ± 140.7	54.8 ± 145.1	97.8 ± 209.2	81.2 ± 164.0	104.2 ± 180.4	6.0 ± 6.8
LK14r	95.0 ± 92.4	211.3 ± 406.4	99.5 ± 121.7	113.2 ± 108.2	112.2 ± 106.8	n/a ± n/a
LK14s	28.4 ± 105.5	22.4 ± 67.6	24.9 ± 121.5	n/a ± n/a	29.6 ± 112.4	12.1 ± 11.1
LK14t	81.2 ± 66.0	132.0 ± 176.0	97.2 ± 99.0	n/a ± n/a	88.2 ± 82.4	18.7 ± 4.6
LK18d	84.3 ± 108.0	87.5 ± 233.3	79.5 ± 102.9	122.2 ± 297.3	97.8 ± 97.8	15.0 ± 11.5
LK18h	n/a ± n/a	55.5 ± 101.9	31.3 ± 48.5	n/a ± n/a	29.1 ± 47.1	6.4 ± 21.0
LK18x	n/a ± n/a	34.2 ± 53.9	29.4 ± 35.4	n/a ± n/a	27.8 ± 36.2	11.4 ± 8.7
LK20g	16.4 ± 28.3	n/a ± n/a	15.1 ± 29.9	n/a ± n/a	18.6 ± 30.0	7.1 ± 4.8
LK20l	32.3 ± 64.1	13.5 ± 18.8	17.3 ± 29.9	n/a ± n/a	20.8 ± 33.2	6.0 ± 3.3
LK22b	27.8 ± 35.7	13.2 ± 20.4	17.5 ± 30.0	n/a ± n/a	25.4 ± 37.0	6.7 ± 4.4
LK25b	9.2 ± 19.4	n/a ± n/a	12.5 ± 33.2	n/a ± n/a	9.5 ± 21.1	8.0 ± 8.1
LK25h	55.1 ± 109.9	15.1 ± 35.9	20.3 ± 115.5	n/a ± n/a	53.1 ± 131.0	7.3 ± 5.4
LK26b	36.1 ± 72.9	73.2 ± 241.6	65.0 ± 141.3	n/a ± n/a	38.2 ± 78.5	10.1 ± 7.6
LK27a	27.9 ± 92.0	20.5 ± 39.2	42.4 ± 158.6	n/a ± n/a	43.6 ± 126.3	9.4 ± 12.9
D25i	10.9 ± 11.3	7.2 ± 8.4	10.2 ± 14.9	n/a ± n/a	10.7 ± 12.7	5.0 ± 3.4
D38j	21.9 ± 32.1	n/a ± n/a	13.5 ± 24.5	n/a ± n/a	19.8 ± 33.8	6.3 ± 3.4
DS20k	19.7 ± 26.9	10.9 ± 21.6	14.5 ± 30.5	n/a ± n/a	19.6 ± 29.5	5.6 ± 2.9
DS20p	64.2 ± 102.5	51.7 ± 98.7	63.0 ± 64.6	85.1 ± 124.4	93.4 ± 128.8	9.4 ± 5.5
DS20z	39.7 ± 28.7	71.1 ± 151.2	41.3 ± 37.8	35.7 ± 47.3	37.6 ± 32.2	7.4 ± 9.5
Median ± interquartile range of ISI during rapid eye movement sleep: *, 26.4 ± 40.1 ms; **, 52.3 ± 83.6 ms; ***, 88.2 ± 142.1 ms; n/a, not applicable.						

The timing of action potentials during individual theta oscillatory cycles showed variation; five out of seven labeled projection cells increased their firing rate during the descending slope of theta cycles around 270° (Fig. 3B,C and Table 1), together with PV‐expressing basket cells. Two neurons were strongly coupled to the trough of theta cycles, similar to bistratified and O‐LM cells (Fig. 3B,C, Table 1 and (Katona et al., 2014)). As a group, most recorded neurons increased their firing rates at and around the trough of theta cycles (Fig. 3B and Table 1). The strength of phase coupling for most cells (*n* = 27/32), including six out of seven labeled projection cells, was relatively high with mean vector lengths between 0.2 and 0.7 (Fig. 3B and Table 1), similar to bistratified and O‐LM cells (Katona et al., 2014).

During SWRs, there was variability in the median number of spikes of the individual cells resulting in mean firing rates ranging between 0.3 Hz and 194 Hz (Fig. 3A and Table 1). Four out of seven labeled projection cells fired no more than two action potentials per SWR, similar to O‐LM cells. In contrast, the other three labeled projection cells fired seven or more action potentials per SWR, similar to bistratified cells (Table 1 and (Katona et al., 2014)). We have compared the firing rates during SWRs to firing rates outside SWRs by calculating a SWR activation index (see methods) with values from −1 (fully inhibited during SWRs) to 1 (activated during SWRs), and zero reporting no difference. We have found positive SWR activation indices for most cells (*n* = 25/32) demonstrating increased firing rates during SWRs as compared to periods outside SWR events (Fig. 4). This included all seven labeled projection neurons (Fig. 3A,B and Table 1). For six out of the seven labeled projection cells the SWR activation values were as high as those calculated for bistratified cells (Fig. 3A,B, Table 1 and (Katona et al., 2014)), with the strongest activation (0.77) observed for DK02Ag. One labeled neuron (D43j) was most similar to O‐LM cells with a SWR activation index of 0.16 and with slightly increased firing rates observed only during SWRs in quiet wakefulness, but not during sleep SWRs (Figs. 3A,B and 4H, Table 1 and (Katona et al., 2014)). The increased firing rates and high SWR activation indices of most projection cells during SWRs matched those of bistratified cells and PV‐expressing basket cells. Moreover, some projection cells demonstrated rhythmic 130–230 Hz firing at ripple oscillation frequency (Fig. 4I), similar to bistratified cells.

The neuropeptide somatostatin is commonly expressed by many neurons in stratum oriens and it may be released by these neurons during high frequency action potential bursts (van den Pol, 2012). We have investigated the occurrence of spike bursts of three or more action potentials with ISIs shorter than or equal to 12 ms during oscillatory network states (Table 3). During theta oscillatory cycles, the probability of such action potential bursts was lower than 20% for the majority of neurons (*n* = 29/32). In fact, 8/32 cells, including two out of seven labeled cells, did not fire such bursts of action potential during theta cycles. This was similar to the burst firing probability of O‐LM but not bistratified cells (Katona et al., 2014). Bursting probability higher than 30% of twelve cells during SWRs was similar to that of bistratified but different from that of O‐LM cells (Katona et al., 2014). High probability bursting like that of bistratified cells was only observed for four projection cells and only during SWRs. We have observed that many cells (*n* = 22/27) fired high frequency bursts with higher probability during SWRs compared to burst probability during theta cycles reported by negative burst probability indices. The burst firing probability of three cells, including two labeled projection cells, increased from less than 1%, during theta, to greater than 88%, during SWRs (D29o, DK02Ag). Seven cells did not fire spike bursts during SWRs, four of which did not fire bursts during theta cycles either. Different probabilities of burst occurrence suggest cell type‐specific conditions and mechanisms underlying synaptic input plasticity (Evstratova et al., 2011) and/or peptide release (van den Pol, 2012).

**Table 3:** Burst Firing Characteristics of GABAergic Neurons in Stratum Oriens

	Burst frequency (Hz)			Burst probability (%)		
Cell	Movement	Slow wave sleep	Index	Theta cycles	SWR events	Index
D29o	0.1	1.0	−0.86	0.8	87.9	−0.98
D43j	0.5	0.3	0.28	5.2	8.0	−0.21
DK02Ag	0.6	2.5	−0.62	0.0	91.7	−1.00
DS17c	0.1	2.2	−0.93	6.7	42.9	−0.73
O82c	1.4	1.6	−0.07	10.1	70.8	−0.75
TV09u	0.0	0.0	n/a	0.0	0.0	n/a
ZsB49b	0.0	0.3	−1.00	0.9	2.9	−0.54
LK06o	3.9	3.6	0.04	36.4	97.7	−0.46
LK10x	0.1	0.1	0.11	0.6	20.0	−0.94
LK13f	0.0	0.7	−0.88	0.0	8.5	−1.00
LK14e	0.3	n/a	n/a	3.4	n/a	n/a
LK14k	0.0	0.1	−1.00	0.8	0.6	0.12
LK14l	0.0	0.3	−1.00	1.0	0.0	1.00
LK14p	0.0	0.3	−1.00	0.5	1.8	−0.53
LK14r	0.0	0.0	n/a	0.0	0.0	n/a
LK14s	0.5	0.4	0.15	5.7	22.2	−0.59
LK14t	0.0	0.0	n/a	0.0	0.0	n/a
LK18d	0.0	0.1	−1.00	0.0	0.0	n/a
LK18h	n/a	0.2	n/a	2.9	0.0	1.00
LK18x	n/a	0.2	n/a	0.0	5.6	−1.00
LK20g	1.8	n/a	n/a	18.6	73.8	−0.60
LK20l	0.2	3.4	−0.90	16.9	95.1	−0.70
LK22b	0.3	2.5	−0.82	7.5	63.8	−0.79
LK25b	5.3	n/a	n/a	54.1	25.0	0.37
LK25h	0.2	1.1	−0.71	3.5	69.3	−0.90
LK26b	0.3	0.2	0.23	2.7	0.0	1.00
LK27a	0.3	1.3	−0.63	1.4	27.0	−0.90
D25i	7.6	7.0	0.05	54.3	88.0	−0.24
D38j	0.9	n/a	n/a	14.4	89.1	−0.72
DS20k	2.0	2.3	−0.06	0.0	93.8	−1.00
DS20p	0.0	0.3	−0.83	0.7	20.4	−0.93
DS20z	0.0	0.1	−1.00	3.2	7.3	−0.38

n/a, not applicable..

### Changes in Neuronal Activity upon Behavioral Transitions

We observed large variability in the behavioral state related mean firing rates and autocorrelograms of neurons recorded in stratum oriens and at the border with stratum pyramidale (Fig. 5).

Almost half of the non‐pyramidal cells (*n* = 12/26) fired with higher rates during movement than during slow wave sleep. The other half were more active during slow wave sleep than during movement, in contrast with O‐LM cells and two bistratified cells; this included four out of seven labeled projection cells (Fig. 5A,B, Table 1 and (Katona et al., 2014)). Firing rates during quiet wakefulness were similar to those during movement. Whenever available, we have calculated mean firing rates during REM sleep and sleep‐wake transition periods (Table 1). One labeled projection cell (DS17c) fired with the highest rate during REM sleep, six fold higher than during movement (Fig. 5B and Table 1). Such variability and behavior dependent differences in firing rates were paralleled by variability and differences in the ISI distributions, with ISI lengths ranging between 9.2 ± 19.4 ms and 189.8 ± 460.3 ms, during movement, and between 7.2 ± 8.4 ms and 270.3 ± 355.4 ms, during slow wave sleep (Table 2). During quiet wakefulness, the ISI distributions were similar to those during movement.

We calculated autocorrelograms to evaluate rhythmicity in the spike trains (Fig. 5B). During movement, most cells (*n* = 17/19), including four out of five labeled projection cells, showed repetitive peaks in their autocorrelograms, on average at every 151 ms, similar to O‐LM and bistratified cells (Table 2 and (Katona et al., 2014)) reflecting rhythmic firing within theta range (5–12 Hz). Such peaks were not present in the autocorrelogram of three cells, including one labeled neuron (D29o). Moreover, if possible, we identified the first peak within the initial 50 ms time range of the autocorrelograms. For most cells, including six out of seven labeled cells, the first peak was between 11 ms and 30 ms (*n* = 19/26) or 8 ms and 10 ms (*n* = 3/26) demonstrating rhythmic firing in the low gamma (30–90 Hz) and high gamma (100–125 Hz) frequency ranges. This was similar to the spiking patterns of both O‐LM and bistratified cells, with only bistratified cells firing in the high gamma frequency range (Katona et al., 2014). The gamma‐ and ripple‐rhythmic spiking of projection cells is probably a result of cooperative interactions of intrinsic properties (Martina et al., 2000; van Hooft et al., 2000; Lien and Jonas, 2003) and the gamma or ripple frequency entrainment of the output of CA1 pyramidal cells.

During slow wave sleep, the autocorrelograms of five out of six labeled cells presented an initial sharp peak, similar to bistratified cells; the autocorrelogram of another cell (D43j) was similar to that of O‐LM cells lacking a sharp initial peak (Fig. 5B and (Katona et al., 2014)). For one labeled cell (TV09u), an insufficient number of spikes were recorded during slow wave sleep and no meaningful autocorrelogram could be generated. We extracted the time points of the first and, if present, the second peak within the initial 50 ms range. We found peaks at less than or equal to 7 ms in twelve out of twenty‐two cells, including three labeled projection cells, demonstrating ripple rhythmic (130–230 Hz) spiking of these cells. This was similar to the sleep‐related firing of bistratified cells, but not O‐LM cells (Katona et al., 2014). One ripple‐rhythmic cell had a second peak in the autocorrelogram at 10 ms demonstrating rhythmicity also in the high gamma range (100–125 Hz). Further, seven ripple‐rhythmic cells, including three labeled projection cells, had secondary peaks in the autocorrelogram in the mid/low gamma range between 11 ms and 30 ms, representing spiking at 30–90 Hz. Seven cells out of twenty‐two, including one labeled projection cell (ZsB49b), fired within the high gamma range with first peaks in their autocorrelograms between 8 ms and 10 ms. Five of these, including one labeled projection cell (ZsB49b), fired also in the low gamma frequency range as evident from a second peak in the autocorrelogram. Three cells, including one labeled projection cell (D29o), were similar to O‐LM cells, as their autocorrelograms indicated gamma rhythmic firing only in the range of low gamma frequency (Katona et al., 2014). Overall, behavioral states divided the group of projection cells into those more active during sleep, similar to PV‐expressing basket cells and some bistratified cells; and others more active during movement, similar to O‐LM cells and some other bistratified cells.

We monitored the burst firing of interneurons during movement and during slow wave sleep (Fig. 5A and Table 3). During movement, many cells (*n* = 24/30), including six labeled projection cells, fired bursts with frequencies lower than or equal to 1.1 Hz, similar to O‐LM cells, but unlike most bistratified cells which fired bursts at higher rates (Katona et al., 2014). The bursting frequency of the other six cells was similar to that of bistratified cells with a maximum of 7.6 Hz (Katona et al., 2014). During sleep, most cells (*n* = 19/28), including four labeled projection cells, still fired bursts with frequencies lower than 1.1 Hz, similar to O‐LM cells (Katona et al., 2014). The higher bursting frequency of six cells was similar to that of bistratified cells (Katona et al., 2014). Overall, most neurons (*n* = 17/23), including five out of six labeled projection cells, fired bursts with higher frequency during slow wave sleep than during movement as reported by their burst frequency indices (more symbols blue on Fig. 5A). In contrast, the bursting frequency of another six cells, including one labeled projection cell (D43j), together with that of two O‐LM cells and two bistratified cells was higher during movement (Katona et al., 2014). Three neurons, including one labeled projection cell (TV09u), did not fire bursts as defined above.

## DISCUSSION

Long‐range projecting and rhythmically discharging GABAergic neurons, most of which have local circuit output within the CA1 area where their somata are located, are rarely encountered and show a remarkable diversity. They complement the GABAergic/peptidergic actions of local axon bistratified and other interneurons in differentially phasing inputs from the CA3 area to CA1 pyramidal cell dendrites during sleep and wakefulness. Together with the rhythmically entrained outputs of glutamatergic pyramidal cells, the fast monosynaptic transmission via long‐range GABAergic projections supports the behavioral‐ and rhythmic state‐dependent coordination of wide‐spread cortical cell assemblies across functionally related brain areas.

### Behavior‐Dependent Activity Patterns of GABAergic Neurons in Freely Moving Rats

We have analyzed in detail rarely encountered GABAergic projection neurons. We have identified similarities and striking differences in the *in vivo* activity of GABAergic projection cells and exclusively local axon interneuron types, e.g. SOM‐expressing bistratified and O‐LM cells (Katona et al., 2014) and PV‐expressing basket cells (Lapray et al., 2012) during natural behavior in freely moving rats. According to their dendritic and axonal projections and molecular composition all labeled neurons reported here are likely SOM‐expressing double projection neurons, which innervate the septum and some cortical areas, or oriens‐retrohippocampal neurons (Jinno et al., 2007), with the exception of one novel CA1 back‐projecting cell (ZsB49b) innervating the DG. Behavior and rhythmic network states determine whether these GABAergic neuron types co‐operate or dissociate their effects in regulating pyramidal cell excitability in the local CA1 network and via long‐range projections in extrahippocampal target areas. Overall, our results demonstrate a spatio‐temporal segregation of action of these GABAergic neuronal subpopulations.

According to soma location, dendritic distribution and molecular composition the CA1‐DG back‐projection neuron ZsB49b is similar to most long‐range projecting GABAergic neuron types and O‐LM cells. However, the dense axonal output of ZsB49b to strata oriens, lacunosum‐moleculare and the DG molecular layer makes it different from any previously documented GABAergic cell. Axonal sprouting of SOM+ neurons has been demonstrated in the dentate molecular layer following pilocarpine induced status epilepticus (Peng et al., 2013). However, the absence of synaptic output to stratum radiatum in contrast to the extensive innervation of stratum oriens ‐ the most striking feature of this cell ‐ is unlikely to be a result of random sprouting resulting from epileptic activity. Contrary to another back‐projection cell with unknown molecular profile reported in the CA1 area targeting the CA3 and to lesser extent the hilar region (Sik et al., 1994), ZsB49b mediates GABAergic action from the CA1 area to the DG. The neuron ZsB49b which we have described in great depth does not resemble any previously published GABAergic neuron in its connections or activity. Based on the location of its axon terminals, it is likely to regulate dendritic integration of inputs from both the CA3 area and the entorhinal cortex while simultaneously assisting in the coordination between the CA1 area and the DG, as evidenced by its strong excitation during SWRs. This single example reveals a unique axonal pattern which together with the exceptional silencing of the neuron during sleep‐wake transitions raises the possibility of ZsB49b being novel cell type. More cells with similar activity and axonal pattern will need to be identified to determine their role and the behavioral correlates of their activity.

Our dataset complements predictions from tetrode recordings of putative GABAergic neurons in strata oriens/alveus (Skaggs et al., 1996; Csicsvari et al., 1999b; Czurkó et al., 2011) by associating cell types and firing patterns. The apparent lack of phase precession in our sample of GABAergic neurons may be due to the rat's slow movement (Maurer et al., 2006; Ego‐Stengel and Wilson, 2007). Moreover, we have validated many of the spike‐timing characteristics determined under urethane anesthesia, although, on average, firing rates are higher under drug‐free conditions. Interestingly, we have found a significant phase shift in the preferential theta oscillations‐related spike‐timing of projection cells, from the descending slope and trough, recorded in freely moving rats, to the trough and ascending slope under anesthesia (Jinno et al., 2007). The absence of spatially tuned, phase‐precessing inputs from pyramidal place cells and perhaps from medial septal GABAergic and cholinergic afferents may contribute to this phase shift under anesthesia.

### Synaptic Organization of Long‐Range Projecting GABAergic Neurons

The main glutamatergic input to horizontal GABAergic cells in stratum oriens is provided by recurrent collaterals of CA1 pyramidal cells (Lacaille et al., 1987; Blasco‐Ibáñez and Freund, 1995; Ali and Thomson, 1998; Kim et al., 2012) via few release sites (Biró et al., 2005). Variable short‐term plasticity (Ali and Thomson, 1998; Losonczy et al., 2002; Biró et al., 2005) and long‐term Hebbian (Perez et al., 2001; Topolnik et al., 2005) and anti‐Hebbian potentiation (Lamsa et al., 2007) have been reported in some interneurons. However, the dynamics of synaptic inputs to identified projection cells have not been characterized. Furthermore, serotonergic and cholinergic pathways from the median raphe (Freund et al., 1990; Varga et al., 2009) and the medial septum (Lovett‐Barron et al., 2014), respectively, are likely to contribute to the modulation of projection cells.

The increased firing rates of long‐range projecting cells along the descending slope and at the trough of pyramidal layer theta cycles is coincidental with the highest probability of firing of active CA1 pyramidal cells (O'Keefe and Recce, 1993; Skaggs et al., 1996). However, the strong theta phase‐modulation of projection cells is unlikely to be explained by CA1 pyramidal cell inputs alone, because, as a population, pyramidal cells show only weak phase‐modulation by theta oscillations (Skaggs et al., 1996). Instead, the spike‐timing of projection cells is probably regulated by rhythmic inhibition via synapses originating from local interneurons and from GABAergic medial septal cells (Freund and Antal, 1988; Takács et al., 2008).

Indeed, horizontal SOM neurons in stratum oriens, including O‐LM cells and projection neurons, are targeted by CR‐ and VIP‐expressing, interneuron‐specific GABAergic IS‐III cells (Acsády et al., 1996; Tyan et al., 2014) and medial septal GABAergic cells (Gulyás et al., 1990). The IS‐III input to O‐LM cells is well‐established on the basis of their spiny dendrites (Chamberland et al., 2010), but it is not known if the smooth‐dendritic projection cells share this highly selective input (Tyan et al., 2014). Unfortunately, the behavior‐dependent activity of both of these GABAergic inputs remains unknown. Some medial septal neurons fire preferentially along the ascending slope and at the peak of hippocampal theta cycles (King et al., 1998; Dragoi et al., 1999). But, whether such neurons are GABAergic and innervate hippocampal GABAergic projection cells have not been tested. Under anesthesia, parvalbumin‐positive septal GABAergic neurons tended to fire either at the peak or at the trough of CA1 theta oscillations (Borhegyi et al., 2004); few septal cells fired along the ascending slope of theta cycles (Viney et al., 2013).

During SWRs, the strong activation of most projection cells may be explained by the highly synchronous population bursts of groups of CA1 pyramidal cells (O'Keefe and Nadel, 1978; Csicsvari et al., 1999a) and a withdrawal of inhibition from the medial septum (Dragoi et al., 1999), perhaps complemented by excitatory medial septal cholinergic input (Lovett‐Barron et al., 2014). A large proportion of medial septal units are significantly suppressed during hippocampal SWRs (Dragoi et al., 1999), but some remain active (Viney et al., 2013). The firing characteristics of septal neurons targeting hippocampal projection cells remains to be demonstrated.

### Network State‐Specific Regulation of Local and Inter‐Regional Activity via Dendritic Inhibition

The observed sharp phase tuning and deep modulation of the firing of GABAergic projection cells by the theta rhythm point to their role in the theta phase‐dependent retrieval of spatial memories (Hasselmo et al., 2002). Coincident with an increasing CA3 input towards the trough on the descending theta slope, projection cells increase firing together with bistratified cells (Katona et al., 2014; Varga et al., 2014), enabling retrieval of stored associations undergoing modification in pyramidal cell dendrites in strata radiatum and oriens of CA1. At the same time, distal apical tuft‐innervating O‐LM cells regulate the glutamatergic inputs arriving from the entorhinal cortex in stratum lacunosum moleculare of CA1 (Varga et al., 2012; Katona et al., 2014). The gamma rhythmic firing of projection cells together with that of bistratified and PV‐expressing basket cells (Katona et al., 2014; Varga et al., 2014) provide inhibitory mechanisms regulating the spike‐timing of CA1 pyramidal cells (Csicsvari et al., 2003; Royer et al., 2012; Lasztóczi and Klausberger, 2016). Via the long‐range GABAergic axons, projection cells transmit this theta/gamma modulation to concurrently active extrahippocampal neurons, whereby they probably promote theta/gamma oscillatory coherence across the connected brain regions (Schomburg et al., 2014).

During SWRs (O'Keefe and Nadel, 1978; Csicsvari et al., 1999a), the strong and rhythmic activation of most long‐range projecting GABAergic cells together with bistratified and PV‐expressing basket cells (Lapray et al., 2012; Hájos et al., 2013; Katona et al., 2014; Varga et al., 2014) entrains the excitability of the dendritic and somatic membranes of CA1 pyramidal cells to ripple frequencies. These three cell types co‐operate by setting windows of minimal excitability of their targets therefore binding the synchronized discharge of pyramidal cells as assemblies (Ylinen et al., 1995; Csicsvari et al., 1999b; Ellender et al., 2010). It is likely that via their long‐range axons these projection cells provide the simultaneous, rhythmic phasing of postsynaptic dendrites in distant extrahippocampal areas. Moreover, SOM‐expressing projection cells and bistratified cells may also contribute to the peptide‐mediated termination of SWRs by releasing somatostatin during their SWR‐related high frequency burst firing (Boehm and Betz, 1997; Tallent and Siggins, 1997)

There is a high degree of functional specialization of hippocampal cell types during sleep and wakefulness. The differentiated release of GABA results in a rhythmic redistribution of inhibition along different pyramidal cell membrane domains necessary for the regulation of pyramidal cell output during behavior.
